# Live-Cell Dynamic Sensing of Cd^2+^ with a FRET-Based Indicator

**DOI:** 10.1371/journal.pone.0065853

**Published:** 2013-06-11

**Authors:** Tai-Yu Chiu, Po-Hsun Chen, Cha-Ling Chang, De-Ming Yang

**Affiliations:** 1 Department of Medical Research and Education, Taipei Veterans General Hospital, Taipei, Taiwan, Republic of China; 2 Institute of Biophotonics, School of Medical Technology and Engineering, Taipei, Taiwan, Republic of China; 3 Biophotonics and Molecular Imaging Research Center, National Yang-Ming University, Taipei, Taiwan, Republic of China; LAAS-CNRS, France

## Abstract

Cd^2+^ causes damages to several human tissues. Although the toxicological and carcinogenetic mechanisms of Cd^2+^ have been previously established, some basic questions on this toxicant remain unclear. In this study, we constructed Met-cad 1.57, a new fluorescent resonance energy transfer (FRET)-based Cd^2+^ indicator, which contains a portion of a Cd^2+^-binding protein (CadR) obtained from *Pseudomonas putida* as the Cd^2+^ sensing key. We produced a human embryonic kidney cell line HEK-MCD157 which stably expresses the Met-cad 1.57 for further investigations. Both fluorescence spectroscopy and FRET microscopic ratio imaging were used to monitor the Cd^2+^ concentration within the living HEK-MCD157 cells. The dissociation constant of Met-cad 1.57 was approximately 271 nM. The function of Ca^2+^ channels as a potential Cd^2+^ entry gateway was further confirmed in the HEK-MCD157 cells. The organelle-targeted property of the protein-based Cd^2+^ indicator directly reveals the nucleus accumulation phenomena. In summary, a human kidney cell line that stably expresses the FRET-based Cd^2+^ indicator Met-cad 1.57 was constructed for reliable and convenient investigations to determine the Cd^2+^ concentration within living cells, including the identification of the entry pathway of Cd^2+^ and sub-cellular sequestration.

## Introduction

In contrast to ions that are important to life, heavy metal/metalloid ions such as Pb^2+^, Cd^2+^, Hg^2+^, and As^3+^ are extremely toxic and not beneficial to living organisms [1]. Molecular mimicry is one of the most accepted concepts that explain the toxic mechanism of these ions [2], [3], including Cd^2+^ [4]. In particular, Cd^2+^ impairs certain biochemical reactions involved in normal physiological functions, thereby causing severe damages in certain targeted cells [1], [4]. Clinically, toxicants are monitored by evaluating the concentrations in the blood or urine through atomic absorption spectroscopy. For Cd^2+^, the acceptable blood Cd^2+^ concentration (BCdC) is approximately 5 µg/L to 10 µg/L [1], [5].

Considering the concepts in molecular and cellular toxicology, some basic questions on Cd^2+^ remain unclear. For example, whether BCdC can fully represent the whole intoxication status in certain human tissues is unknown. Various membrane proteins that have been proposed as transport molecules for Cd^2+^ entry is yet to be confirmed [6], [7], [8]. For metal ion trafficking, the sub-cellular sequestration and accumulation of Cd^2+^ have been observed in several organelles such as the mitochondria or nucleus [9], but have not been completely elucidated. Conclusions for these issues cannot be easily obtained partially because of the lack of suitable live-cell tools that are specific for cytosolic and sub-cellular Cd^2+^ sensing [10], [11]. Ideally, the patch-clamp electrophysiological method can precisely determine the specific ionic current flux into or out of living cells, and thus has been used as the first choice for cytotoxic Cd^2+^ investigations [6]. However, the ion channel recording can neither reveal the Cd^2+^ currents chronically (e.g., at least 1 h to 2 h long-term recording), not monitor the sub-cellular dynamics of Cd^2+^. To obtain accurate information on the succeeding sequestration and the bio-magnification of Cd^2+^ after cytotoxic entry, appropriate live-cell sensing approaches for intra/sub-cellular Cd^2+^ monitoring have been attempted. Reliable method that can directly observe the Cd^2+^ toxicity status within intact cells should be developed because this method can potentially help to identify the agents that can efficiently remove Cd^2+^ from the human body.

In this study, we established a fluorescent protein-based Cd^2+^ indicator, Met-cad 1.57, which is applicable for both intracellular and sub-cellular Cd^2+^ detections. The sensing element is CadR, a dimer protein responsible for the Cd^2+^ resistance of *Pseudomonas putida* [12]. The sensing ability of Met-cad was characterized for the content monitoring of Cd^2+^. The human embryonic kidney (HEK) cells that stably express Met-cad 1.57 (HEK-MCD157) were produced for further investigations.

## Results

### Development of Met-cads

**Figure 1:**
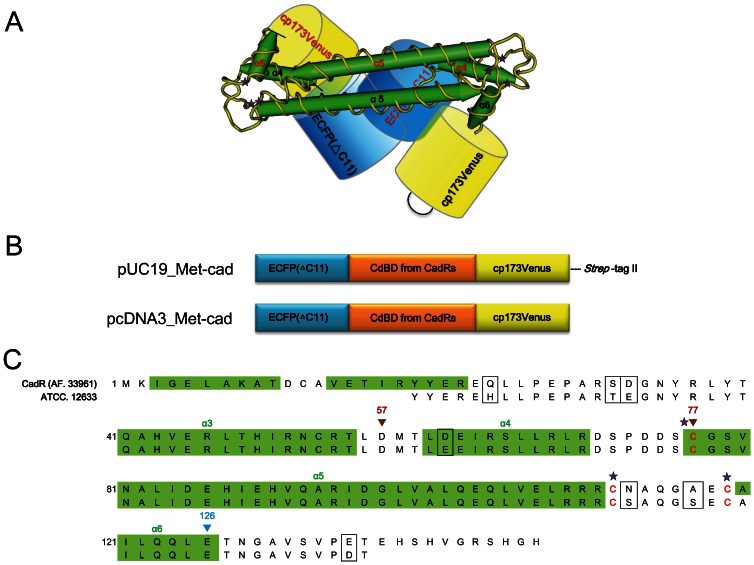
Design of fluorescent resonance energy transfer (FRET)-based Cd^2+^ indicators. (A) Proposed protein structure of Met-cad, a FRET-based Cd^2+^ indicator, which contains the FRET pair ECFP (ΔC11) (blue) and cpVenus (yellow) as well as CadR (green) with the Cd^2+^ sensing site (yellow with 3 stars). The ZntR structure (PDB ID code 1Q08) was used as a model for CadR [39]. (B) Genetic map for Met-cad constructs. For bacterial expression, pUC19 is the major vector that contains the FRET pair gene and the Cd^2+^-binding domain, CdBD. For the large-scale preparation of Met-cad, *Strep*-tag was ligated at the end of the constructs. For the mammalian cell expression, pcDNA3 vector was used to carry the Met-cad construct into the cells or sub-cellular compartments. (C) Detailed protein sequence of CadR designed as the Cd^2+^-sensing center to construct Met-cads. The *P. putida* (ATCC, 12633) was so far the only available source for CadR in our laboratory. The proposed CadR protein fragment from ATCC 12633 was therefore compared with the known CadR sequence from AF 33961 (*P. putida* 06909) as previous reported [12]. These two are found to be highly conserved. For Met-cad 1.57, the protein sequence begins at the 57^th^ residue (red inverted triangle), whereas the protein sequence for Met-cad 1.77 begins at the 77^th^ residue (red inverted triangle). Both constructs ended at the 126^th^ residue (blue inverted triangle). The three stars labeled cysteine residues of each CadR unit are the proposed Cd^2+^ binding sites under the conformation of intermolecular dimer [12].

From our recent report, we proposed that a certain part of MerR-like protein is a possible metal ion sensing toolbox that produces an identical metal ion indicator, such as Met-lead with PbrR [13]. In this study, we selected CadR, another MerR family member, to design Met-cad, a Cd^2+^ indicator (Figure 1). The proposed dimer structure of a FRET-based Cd^2+^ indicator is shown in Figure 1A according to previous report about the structure property of CadR [12]. The FRET event can occur if ECFP (ΔC11) and cpVenus (as an effective FRET pair) can be driven close together upon the CadR-Cd^2+^ binding. Figure 1B reveals the genetic maps of Met-cads designed for bacteria and mammalian cell expressions by using pUC19 and pcDNA3 vectors, respectively. Two versions of Met-cads were constructed: Met-cad 1.57, with the protein sequence from residue 57 to 126; and Met-cad 1.77, with the protein sequence from residue 77 to 126 (Figure 1C). The following sub-cellular targeted constructs for mammalian cell were produced with specific organelle-localized sequences.

### FRET Spectra of Met-cads for Cd^2+^ Sensing

**Figure 2:**
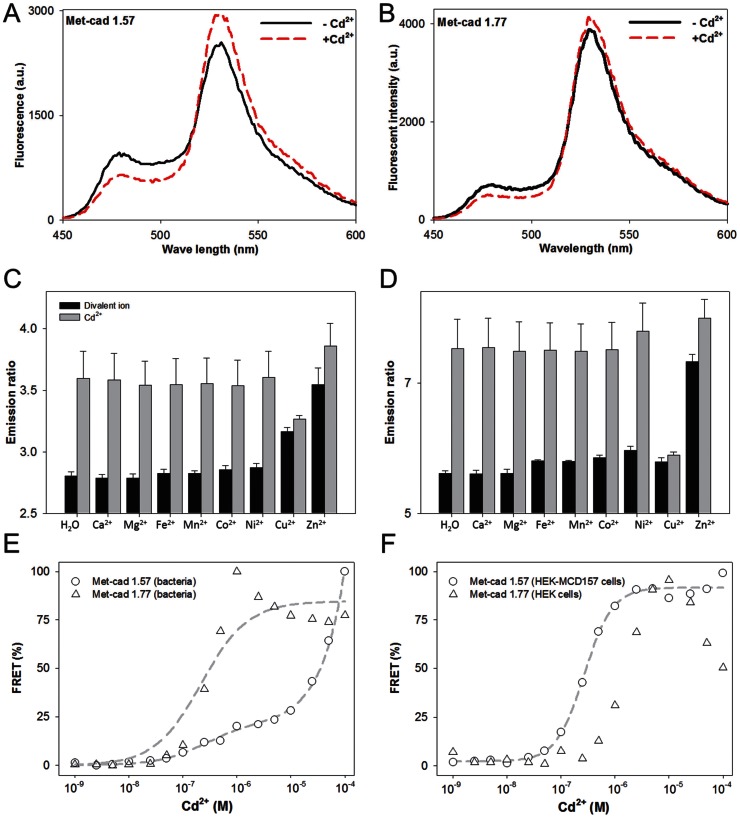
Spectral analysis of Met-cads. (A and B) Pattern changes in fluorescence spectra from bacterial Met-cads 1.57 (A) and 1.77 (B) between the control group [(–)Cd, solid line] and Cd [(+)Cd, 100 µM of Cd^2+^; dashed line]. The YFP/CFP (emission ratio) values of Met-cads range from 520 nm to 550 nm for YFP and 460 nm to 490 nm for CFP, which correspond to the fluorescent resonance energy transfer (FRET) status. (C and D) Ion selectivity of Met-cads 1.57 (C) and 1.77 (D) to Cd^2+^ (1 µM) was tested (compared with the emission ratios) on various ions (1 µM): Ca^2+^, Mg^2+^, Fe^2+^, Mn^2+^, Co^2+^, Ni^2+^, Cu^2+^, and Zn^2+^ without (black bars) or with (gray bars) Cd^2+^. (E and F) Titration curves of bacterial (E) and mammalian (F) Met-cads, 1.57 (circle) and 1.77 (triangle). Normalized emission ratio data presented as FRET percentage (%) of Met-cads were recorded under different Cd^2+^ concentrations (10^–9^ M to 10^–4^ M). The fitting curves (dash lines) of bacterial Met-cads 1.57 and 1.77 as well as the mammalian Met-cad 1.57 are also shown.

The spectral patterns of ECFP (ΔC11) [fluorescent intensity (FI) at 475 nm] and cp173Venus (FI at 535 nm) from bacterial and mammalian Met-cad proteins between the control solution (without Cd^2+^; solid line in Figures 2A, 2B, and S1) and the Cd^2+^ solution (100 µM, dashed line in Figures 2A, 2B, and S1) are shown. The YFP/CFP FRET emission ratio of Met-cads was used to present the Cd^2+^-sensing capacity qualitatively. Such emission ratios can be obtained from the original spectral data (e.g., Figure 2C and 2D). The increase in FI of YFP and the decrease in FI of CFP indicate the occurrence of a successful Cd^2+^-dependent FRET event and correspond to the Cd^2+^-sensing ability of Met-cads (Figures 2A, 2B, and S2).

For sensing specificity, the Met-cads were then challenged with metal ions other than Cd^2+^. The emission ratio changes in Met-cads (1 µM) of the control group (H_2_O) or selected divalent ions (1 µM), such as Ca^2+^, Mg^2+^, Fe^2+^, Mn^2+^, Co^2+^, Ni^2+^, Cu^2+^, and Zn^2+^ (black bars in Figures 2C to 2D) are shown to present the ion selectivity of Met-cads on Cd^2+^ sensing. To demonstrate the competitive ability of Met-cads (gray bars in Figure 2C to 2D), Met-cads were also treated with the selected ions and Cd^2+^. The increase was approximately 0.8 (2.8 to 3.6, about 1.29 fold increase) for Met-cad 1.57 (Figure 2C) and 2.6 (5.2 to 7.8, 1.5 fold increase) for Met-cad 1.77 (Figure 2D). There are some adverse effects of Zn^2+^ and Cu^2+^ on the Cd^2+^ sensing (Figure S2). Even though Zn^2+^ alone can induce increase of FRET ratio (Figure S2A), Cd^2+^ still can cause additional significant FRET ratio increase under the existence of equal content of Zn^2+^ (Figure 2C for Met-cad 1.57 and 2D for Met-cad 1.77). Therefore the results here indicated that Cd^2+^ can induce a specific increase in FRET ratio without the influence of other divalent ions, except for Cu^2+^ (Figure S2B).

To determine the detailed sensing capability of Met-cads, the ratios under the spectral platform were further obtained and analyzed under various Cd^2+^ concentrations (10^–9^ M to 10^–4^ M). The titration curves (Cd^2+^ concentrations versus emission ratios; curve fitted as described in the Materials and Methods section) of bacterial (Figure 2E) as well as mammalian (Figure 2F) Met-cad Met-cads 1.57 (circle) and 1.77 (triangle) are shown in Figures 2E and 2F. *K*_d_s of the bacterial Met-cad 1.57 and Met-cad 1.77 were 250 (calculated by one-site saturation with a nonspecific factor Ns = 796460) and 221 nM (calculated by one-site saturation without Ns), and that of mammalian Met-cad 1.57 was 271 nM (calculated by Hill equation).

The length of Met-cad 1.57 is longer than that of Met-cad 1.77 containing additional α4 helix. This causes spatial difference between ECFP (ΔC11) and cpVenus among these two sensors. Various basal FRET value and different FRET response can be acquired in the presence of Cd^2+^. The fact that the pattern of FRET ratio values in Met-cad 1.77 dropped when the concentration of Cd^2+^ is rising higher than 10^−6^ M (triangle in Figures 2E and 2F) is still hard to be explained. We finally chose Met-cad 1.57 for the rest of the experiments.

As described above in Introduction that both MerR including CadR (as well as PbrR [13]) and MerR-based FRET sensors essentially form dimer to construct functional Cd^2+^ binding (similar for Pb^2+^ binding in PbrR). It might raise a concern that such structure could disturb the FRET event for real sensing. The result that we still can observe the FRET event after adding Cd^2+^ (in Met-cads, Figure 2) or identical metal ion (Pb^2+^ in Met-leads in [13]) answered this question.

From experimental data in Figure 2E (dashed line alone with circle), there did exist a novel nonspecific factor (as described above) correlated to the dramatic increase of FRET ratio under high concentration of Cd^2+^ (from 5×10^−5^ M) in bacterial Met-cad 1.57. Alternatively, a stable-expressed cell line HEK-MCD157 was adapted to express large amount of Met-cad protein without the tag to perform the curve fitting (dashed line alone with circle in Figure 2F). The FRET ratio of mammalian 1.57 was saturated (around 5×10^−6^ M), significantly different from the increase pattern shown in bacterial Met-cad 1.57. Base on genetic sequence, the *Strep*-tag could be one of the main differences between bacterial and mammalian Met-cad 1.57, in addition to the two different expression systems (perhaps more complicated modifications like glycosylation make them different). More experiments need to be done in the future.

### Live-Cell Spectral Sensing and Visualization of Cd^2+^

**Figure 3:**
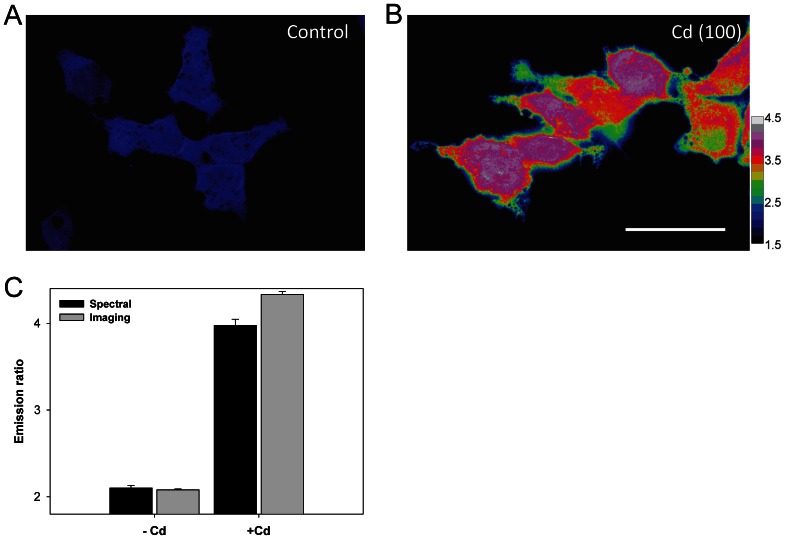
Live-cell fluorescent resonance energy transfer (FRET)-based Cd^2+^ sensing within human embryonic (HEK)-MCD157 cells. (A to B) Representative FRET ratio images of Met-cad 1.57 stably expressed in cells (HEK-MCD157) without (A, Control) or with 100 µM of Cd^2+^ incubated for 2 h [B, Cd (100)]. The ratio color bar ranges from 1.5 to 4.5 (color change sequence: black, blue, green, yellow, orange, red, pink, gray, and white). Scale bar = 10 µm. (C) YFP/CFP emission ratios of living HEK-MCD157 cells. The original data were obtained from fluorescent spectroscopy (Spectral, black bars) or FRET ratio imaging (Imaging, gray bars). These HEK-MCD157 cells were treated without [(–) Cd] or with 100 µM of Cd^2+^ [(+) Cd] for 2 h.

For the easy handling of live-cell Cd^2+^ sensing with an acceptable throughput scale, HEK-MCD157, a Met-cad 1.57 that is stably expressed in the HEK293 cell line, was obtained (see Material and Methods section). Considering Met-cad 1.57 under a visualization aspect (from blue to pink and gray under the ratio color bar defined from 1.5 to 4.5), the homogeneous blue (ratio ≈ 2) cytosolic space in the control cells (Figure 3A) and the pink areas (ratio ≈ 4.35) in the cells that were exposed to 100 µM of Cd^2+^ (Figure 3B) clearly and directly reveal the real status of cytosolic Cd^2+^ contents. Figure 3 shows that the ratio from both spectral and imaging systems may be comparable for further usage. We used ionomycin to test the sensing range of Met-cad 1.57 *in vivo* since this chemical agent can be used as ionic carrier into living cells [13], with a ratio that ranges from 1.86 (basal without Cd^2+^) to 4.29 (100 µM of Cd^2+^), thereby yielding approximately 2.3-fold (data not shown).

### Monitoring the Cytosolic Entry of Cd^2+^

**Figure 4:**
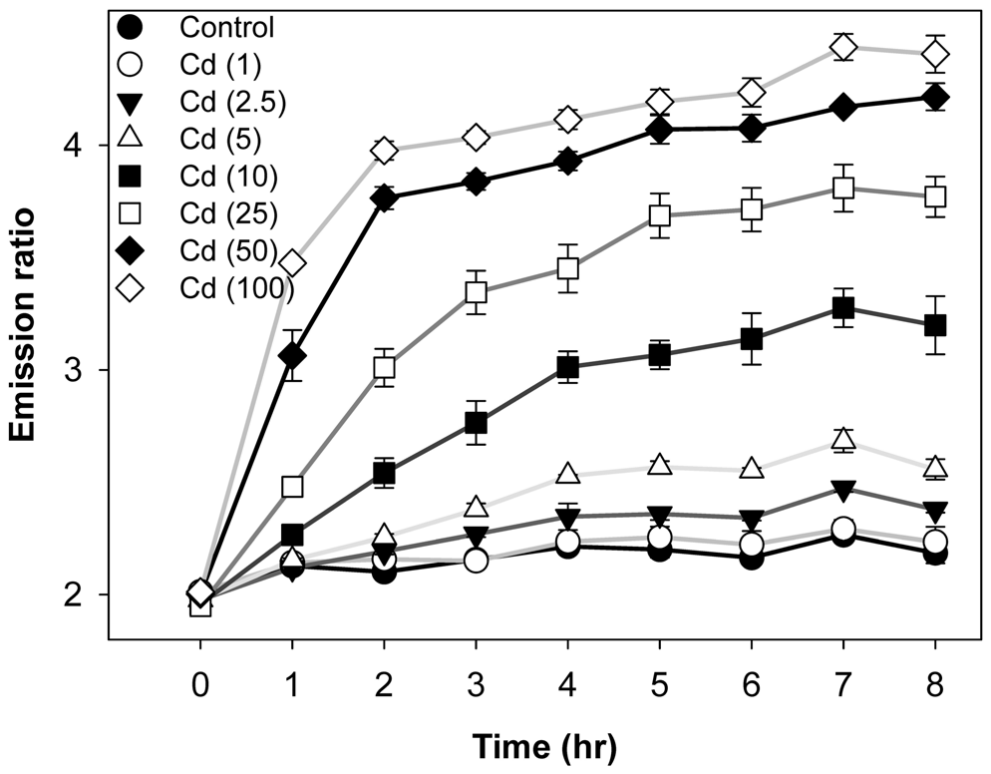
Content monitoring of intracellular Cd^2+^. Human embryonic kidney (HEK)-MCD157 cells cultured in a 96-well plate treated with Cd^2+^ from 1 h to 8 h under various concentrations: control (closed circle), 1 µM (open circle), 2.5 µM (closed inverted triangle), 5 µM (open triangle), 10 µM (closed rectangle), 25 µM (open rectangle), 50 µM (closed diamond), and 100 µM (open diamond). The emission ratio data were obtained by fluorescent spectroscopy.

HEK-MCD 157 cells were used in the following live-cell experiments to monitor the intracellular Cd^2+^ content with the passage of time (recorded every 1 h to 8 h of exposure) in various concentrations of extracellular/environmental Cd^2+^ [from low (1, 2.5, and 5 µM) to high concentrations (10, 25, 50, and 100 µM)]. Figure 4 shows that 1 h to 2 h of relatively high dosage treatments [10 µM (closed square); 25 µM (open square); and 50 µM (closed diamond)] can significantly increase the emission ratio compared with the basal level. By contrast, low concentration (e.g., 2.5 µM) exposure with sufficient incubation time, such as 7 h, also caused a significant increase in ratio (inverted triangle in Figure 4). Although the Cd^2+^ level did not change throughout the 8 h incubation period at 1 µM of Cd^2+^ (empty circle in Figure 4), such trace amounts of Cd^2+^ for a longer exposure time (at least 24 h) can be sensed by Met-cad 1.57 (imaging data shown in Figure S3). The ratios reached 2.401±0.075 and 2.687±0.075 after 24 and 48 h of treatments, respectively, which are significantly higher than those of the basal level (2.013±0.001).

### Ca^2+^ Channels are Involved in the Cytosolic Entry of Cd^2+^

**Figure 5:**
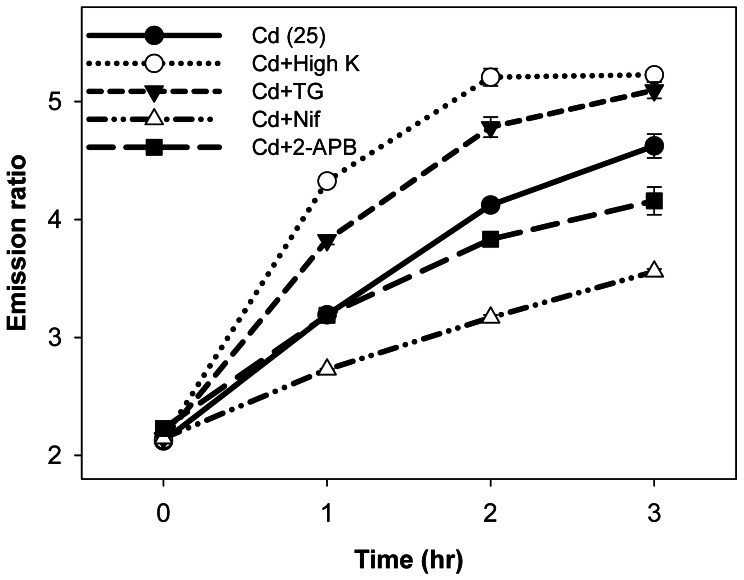
Cd^2+^ Uptake Mechanism. Human embryonic kidney (HEK)-MCD 157 cells seeded in a 96-well culture plate without [Cd (25), closed circle with solid line] or with different additional agents: high K^+^ buffer (Cd+High K, 150 mM; open circle with dotted line), thapsigargin (Cd+TG, 1 µM; closed inverted triangle with dashed line), nifedipine (Cd+Nif, 50 µM; open triangle with dashed-dotted line), and 2-APB (Cd+2-APB, 25 µM, closed rectangle with long dashed line).

We investigated the entry routes of Cd^2+^ into the HEK-MCD157 cells. The data show that the ratios reached 3 after 2 h of incubation at 25 µM of Cd^2+^ (Figure 4). This condition was used as the experimental background to test the possible transport mechanisms for Cd^2+^ entry (Figure 5). Either the agonists (stimulants of Ca^2+^ channels) or the antagonists (blockers of Ca^2+^ channels) were applied (30 min prior to the Cd^2+^ recording, Table S1) as the pharmacological strategy to challenge the potential gateways for Cd^2+^ entry. Figure 5 shows that the activation of different types of Ca^2+^ channels [high K^+^ buffer for voltage-gated channels (open circle with dotted line); thapsigargin (TG) for storage-operated channels (inverted triangle with dashed line)] significantly enhanced Cd^2+^ entry. These channels can be blocked using the corresponding antagonists (nifedipin for voltage-gated Ca^2+^ channels, open triangle with dashed-dotted line; 2-APB for storage-operated Ca^2+^ channels, closed rectangle with long dashed line), which significantly affected Cd^2+^ entry (Figure 5) in agreement with the finding from previous report [6]. The results suggested that various types of Ca^2+^ channels are involved in Cd^2+^ entry.

### Sub-Cellular Sequestration of Cd^2+^

**Figure 6:**
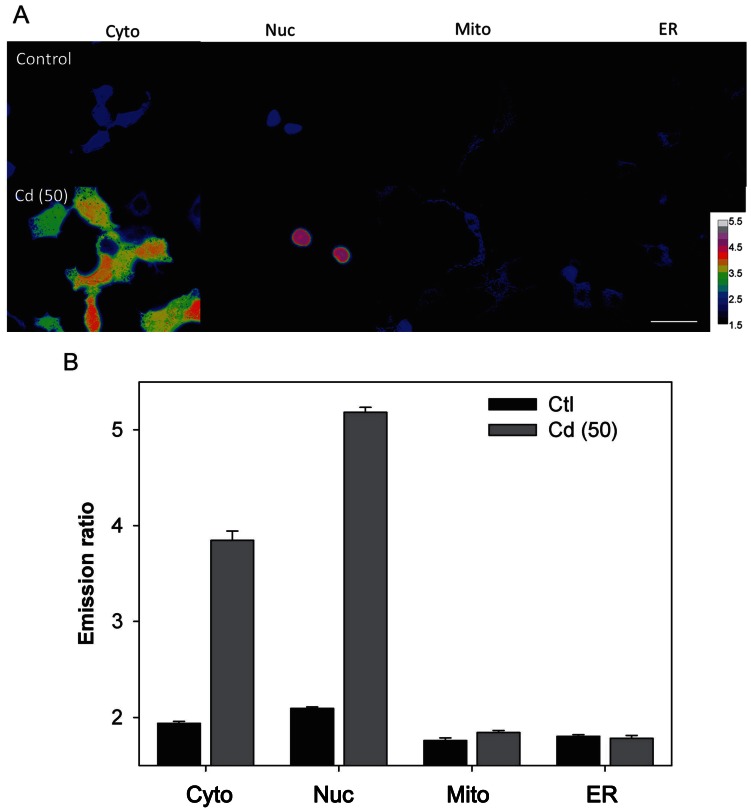
Sub-cellular Cd^2+^ accumulation. (A) Representative images of Met-cads with various organelle-targeted versions (Cyto for cytosol; Nuc for nucleus; Mito for mitochondria; ER for endoplasmic reticulum) without (Control) and with 2 h incubation period at 50 µM of Cd^2+^ [Cd (50)]. The color bars show the ratio that ranges from 1.5 to 5.5. Scale bar = 10 µm. (B) The emission ratios from different sub-cellular compartments are shown without (Control in black bars) and with 50 µM of Cd^2+^ [Cd (50) in gray bars].

The organelle-targeted versions of Met-cad 1.57 were used to visualize the sub-cellular localization of Cd^2+^ that successfully entered the cell (Figure S4). Representative ratio color images of cytosolic (Cyto) and organelle-targeted Met-cad 1.57 within living cells (nucleus, Nuc; mitochondria, Mito; endoplasmic reticulum, ER) are shown in Figure 6A without (control) or with 2 h of exposure to 50 µM of Cd^2+^ [Cd (50)]. The bar figures in Figure 6B indicate the different sub-cellular Cd^2+^ levels. A significant increase in Cd^2+^ contents in the nucleus after 2 h of incubation was observed (higher than the cytosolic Cd^2+^ level). In contrast to the cytosolic and nuclear spaces, no further increase in ratio was found within the mitochondria and ER under the same treatment condition.

## Discussion

The selection of a suitable sensing key is considered one of the difficult processes in manufacturing protein-based metal ion indicators [13], and only three have been successfully manufactured, specifically Ca^2+^ [14], [15], Zn^2+^ [16], and Cu^+^ [17]. In this study, we constructed additional ones for Pb^2+^ [13] and Cd^2+^. Bacteria such as *Cupriavidus metallidurans* CH34 and *P. putida* survive in extremely harsh environments by evolving protective anti-metal systems [12], [18]. MerR is one of the specific metal-binding systems that comprise five major members: PbrR, CadR, MerR, CueR, and ZntR. We initially produced the MerR-based indicator (Met-lead 1.59) for live-cell Pb^2+^ sensing with PbrR used as the metal-binding domain [13]. In this study, part of the CadRs was designed as the Cd^2+^-binding domain to create Met-cads, the second MerR-based indicator for Cd^2+^ (Figure 1). Such serial MerRs can be used as a novel sensing toolbox for individual metal ion sensing (the comparisons on sensing part between Met-lead 1.59 and Met-cad 1.57 are shown in Figure S5). Although we preliminarily established some of the other MerR-related metal ion biosensors, such as those for Cu^+^ (CueR, unpublished data) and Zn^2+^ (ZntR, unpublished data), but those for Hg^2+^ (MerR) have not been developed yet. Further studies should be conducted to prove this concept.

Compared with the currently available fluorescent indicators for live-cell Cd^2+^ sensing, such as the commercial fura-5F [19], fluo-3 [20], and even indo-1, which are originally designed for Ca^2+^ sensing, the sensors can only detect the presence of Cd^2+^ that is incubated at 30 µM for 24 h, and Cd^2+^ cannot be distinguished from Ca^2+^. New developed chemical indicators own much better sensing ability especially the ionic selectivity to distinguish Cd^2+^ from Zn^2+^ [21]–[23]. These indicators tend to be ratiometric within suitable emission wavelength [22], [23] for popular usage on conventional fluorescent imaging. Among these three, CadQM developed by Taki et al., might be the most convincing one for live-cell sensing among these developed chemical-based Cd^2+^ indicators [21]–[23]. The basal level of a Cd^2+^ ratio dye CadMQ is about 0.75 increased to 0.84 under 5 µM Cd^2+^ exposure for 3 hrs (about 1.12 fold increases). (Met-cad: 2.1±0.02; reached to 2.5±0.06, about 1.19 fold increase, shown in Figure 4), and decreased its ratio to 0.74 upon TPEN chelation [23] (we also did this chelation test to confirm, ratio changed back to 2.2±0.03). However, the sensing ability of CadMQ was also affected by Zn^2+^ and Cu^2+^ (Zn^2+^ and Cu^2+^ did have certain influence on Met-cad sensing as shown in Figure S2) and even Hg^2+^. The rest limitations of these chemical dyes are probably the inability to fully organelle targeting specifically (Figures 6, S4) as well as within whole animal sensing (whole animal genetically expressed protein-based biosensor, e.g. Met-cad, can be archived through transgenic way in the near future).

In this study, the newly developed Met-cad 1.57 exhibits a sub-µM level of *K*_d_ (250 nM), relatively high sensitivity, and good selectivity without the influence of Ca^2+^ as well as organelle-targetable characteristic. Met-cad 1.57 can sense the accumulation of Cd^2+^ at very low concentrations (1 µM) for 24 and 48 h of incubation periods. The non-cytotoxicity at 1 µM [19] requires further investigations.

Although the gating mechanisms of Cd^2+^ entry are considered as different types of Ca^2+^ channels [24], [25], [26], divalent metal-ion transporter (DMT1) [8], [27], [28], and Zn^2+^ transporter (ZIP8/14) [29], [30], the conclusions on the Cd^2+^ entry pathway remain controversial. We used the HEK-MCD157 cells to confirm that Ca^2+^ channels are one of the corresponding gates for Cd^2+^ entry (Figure 5). The other candidates such as DMT1 and ZIP8/14 would be tested in future investigations.

Among the available live-cell sensing methods, including chemicals and proteins, the protein-based indicators are probably one of the most promising indicators because of the organelle-targeting and genetic-encoding ability. These indicators can monitor the target biomolecules in the intra/sub-cellular space within living cells and in organisms as a whole [10], [14], [15]. In the current study, we produced three kinds of organelle-targeted versions of Met-cads for the nucleus, mitochondria, and ER. Cd^2+^ accumulation was found inside the nucleus within 2 h of exposure, but not in other sub-cellular compartments (Figure 6). Such Cd^2+^ accumulation within the nucleus was higher than that in the cytosolic Cd^2+^ contents. Cd^2+^ has been considered as a human carcinogen for years [31], [32], [33]. Thus, the nuclear transport mechanism of Cd^2+^ should be investigated in the future because this phenomenon may be correlated with the carcinogenic property of Cd^2+^. The data from sub-cellular localization of heavy Cd^2+^ metal provide valuable insight on heavy metal ion toxicology.

In addition, we established a live-cell system that stably expresses Met-cad 1.57 for numerous benefits. For example, large amounts of Met-cad proteins were provided to help reveal the hidden problem of *Strep*-tag in Met-cads. The live-cell system can be seeded in multi-well cell culture plates to perform easy median-scale experiments such as titration (Figure 2F), time-dose response (Figure 4), and Ca^2+^ channel tests (Figure 5). Furthermore, the live-cell system can be applied in real high-throughput platforms in either spectral or imaging modality with a 96−/386-well plate for more advanced investigations on the molecular and cellular toxicology of Cd^2+^. The possible effective agent(s) for therapeutic usage on Cd^2+^ poisoning would be possibly established through a large-scale screening.

In summary, we produced Met-cad 1.57, another MerR-based indicator for Cd^2+^, according to the FRET strategy. This live-cell Cd^2+^ indicator was further incorporated into a stable HEK cell line or to become organelle-targetable for advanced research on sub-cellular Cd^2+^ dynamics. These constructs and cell line can be further applied in several investigations on the molecular and cellular toxicology of Cd^2+^.

## Materials and Methods

### Chemicals

All reagents were purchased from Sigma Chemical Co. (St. Louis, MO, USA) unless indicated otherwise. Most of the reagents for molecular cloning, such as restriction enzymes, ligases, and others, were from New England BioLabs. The polymerase chain reaction kit and the primers used were purchased from Finnzymes Oy (Finland) and Mission Biotech (Taiwan), respectively. The DNA extraction kit was purchased from Viogene.

### Gene Construction and Sub-Cellular Targeting of Met-cad

According to the gene alignment program Kalign (EMBL-EBI) and the structure prediction PredictProtein [34], the gene fragments of CadR from *P. putida* [American Type Culture Collection (ATCC, Rockville, MD, USA) 12633] were compared with AF 33961 [12] and were cloned as the Cd^2+^-binding domain (CdBD) for the Cd^2+^-sensing. The protein sequence map (Figure 1A) shows that the CdBDs, ECFP (ΔC11), and cp173Venus were ligated together [13], [15] as the Met-cads, which were further cloned into pcDNA3 vector (Invitrogen) to form pcDNA3_Met-cads (Figure 1B). Organelle-targeted versions of Met-cad 1.57 were constructed by adding specific targeted sequences as previously reported, such as the sequences in the nucleus (two tandem repeats of DPKKKRKV derived from the simian virus large T-antigen) [35], mitochondria (four tandem repeats of SVLTPLLLRGLTGSARRLPVPRAKIHSL derived from the precursor of subunit VIII of human cytochrome C oxidase) [36], and endoplasmic reticulum (calrericulin signal sequence LLSVPLLLGLLGLAVA with the retention sequence KDEL) [37], [38].

### Cell Culture, Transfection, and Stable Expression of Met-cad in HEK-MCD157 Cell Line

HEK293 cells from the ATCC were cultured in 22 mm coverslip and transfected [13] with pcDNA3_Met-cad 1.57 or other organelle-targeted versions of Met-cad 1.57 for live-cell fluorescent resonance energy transfer (FRET)-based microscopic ratio imaging.

The HEK-MCD157 cells, which stably express Met-cad 1.57, were produced through antibiotic (G418) selection. These cells were seeded in 96-well culture plates (COSTAR 3599, Corning, USA) for fluorescent spectroscopy or cultured as described for FRET ratio imaging.

### Fluorescent Spectroscopy

The fluorescent spectrum of Met-cad 1.57 and its YFP/CFP emission ratios were obtained using a spectrophotometer (Infinite M1000, TECAN, Switzerland) as previously reported [13]. The band settings for CFP and YFP are 460 nm to 490 nm and 520 nm to 550 nm, respectively.

### Live-cell FRET-based Microscopic Ratio Imaging

The live cell Cd^2+^ imaging was performed by acquiring the emission ratios under the FRET imaging platform. The microscope (Axio D1, Zeiss, German) was equipped with a 40× objective lens (NA = 0.75). A metal fluorescent light (X-Cite, Lumen Dynamics Group Inc., Canada) filtered with a band-pass filter (426 nm to 446 nm) and a dichroic mirror (442 nm) was used as the excitation light source. Ratio images of the Met-cads within living cells that contain the fluorescent signals of CFP (483/32 nm) and YFP (542/27 nm) were captured using a dual charge-coupled device camera system (ORCA-D2, Hamamatsu, Japan) with HCImage software (Hamamatsu). Ratio imaging was further displayed in a ratio color plate using the ImageJ analysis software (NIH).

For the experiment using ionomycin as ionophore to transport Cd^2+^ across cell membrane into cytosol, a Ca^2+^ free buffer containing 150 mM NaCl, 5 mM KCl, 1 mM MgCl_2_, 10 mM HEPES, and 5 mM D-glucose (pH 7.4) with 5 µM of ionomycin was used to be without or with a defined Cd^2+^ solution (100 µM) to cells for 10 min before taking ratio imaging [13].

### Extraction of Met-cad Protein, Titration, and Selectivity Tests

The expression and purification of bacterial Met-cad proteins were performed using the same strategy as previous report, the *Strep*-tag [13]. In this study, the *Strep*-tag was used for the following protein purification instead of applying His-tag to avoid non-selective metal ion interaction, i.e. the binding of Ni^2+^ to His-tag. Actually Ni^2+^, as predicted, did not influence the sensing ability of Met-cad with *Strep*-tag (Figures 2C and 2D on Ni^2+^). The only concern about *Strep*-tag would be its possible involvement on the nonspecific factor found in bacterial Met-cad 1.57 (Figure 2E). The mammalian Met-cad 1.57 without the *Strep*-tag stably expressing in the HEK cell line was therefore alternatively prepared by partial purification to test this issue. Briefly, the supernatant with a large amount of Met-cad protein expressed from HEK-MCD157 cells after sonication were further centrifuged to remove the cell debris from the lysate. The data from mammalian source was found to be well fitted on Hill equation.

Simple quantification of these protein indicators was performed prior to titration and selectivity experiments (from 10^–9^ M to 10^–4^ M of Cd^2+^) by fluorescent spectroscopy. To estimate the dissociation constant (*K*_d_) of Met-cads expressed from bacterial sources or HEK-MCD157 cells, the ratio data from the titration experiments were normalized as percentages of FRET relative to the maximal difference of the emission values (*R*). The fitting curve was further analyzed using the following equations: (1) One-site saturation with a nonspecific factor, [Cd^2+^] = [(*R*_max_ × *R*)/(*K*_d_+*R*)]+*n* × *R* (bacterial version); (2) Hill equation [14], [Cd^2+^] = *K*_d_ [(*R* – *R*_min_)/(*R*_max_ – *R*)^(1/*n*)^ (mammalian version), where *n* is the nonspecific binding coefficient. For the selectivity tests (specificity and competition), 1 µM of divalent ions (such as CaCl_2_, MgCl_2_, FeSO_4_, MnCl_2_, CoCl_2_, NiCl_2_, CuCl_2_, and ZnCl_2_) were used with or without equal CdCl_2_ concentrations (1 µM).

### Data Analyses and Statistics

All experiments were conducted thrice with at least three different sets of cell samples. Data gathered from the different batches were integrated to calculate the emission ratio. Significant changes were considered significant at the 0.05 level by ANOVA (SPSS statistics 19, IBM) described previously [13].

## Supporting Information

Figure S1 **Spectral Pattern changes of mammalian Met-cad 1.57 between the control group [(–)Cd, solid line] and Cd [(+)Cd, 100 µM of Cd2+; red dashed line].**
(TIFF)

Figure S2 **Effects of Zn^2+^ and Cu^2+^ on the spectral pattern of Met-cad 1.57.** The spectral patterns of Met- cad 1.57 to Zn ^2+^ (A, 1 µM in dotted line) and to Cu^2+^ (B, 1 µM in dashed line; 10 µM in dashed-dotted line; 100 µM in dotted line) compared with the control without specific ions (Apo in solid line).
(TIF)

Figure S3 **Long-term monitoring of intracellular Cd^2+^ content.** The representative images of cells expressing Met-cad for monitoring intracellular content of Cd^2+^ are shown in YFP (top), CFP (middle), and ratio (bottom) with 8, 12, 24, and 36 hours of incubations (1 µM). The color bars show the ratio that ranges from 1 to 5.
(TIF)

Figure S4 **Sub-cellular targeting of Met-cads within cells.** On top, the representative confocal images (LSM 5 Pascal, Zeiss, Germany, with a 63× oil objective, NA = 1.4, on an inverted microscope, Axiovert 200M) [1], [2] of Met-cads (EX: 458 nm; EM: LP475 nm) of cells transfected with various organelle-targeted versions (Met-cad-nuc for nucleus; Met-cad-mito for mitochondria; Met-cad-ER for endoplasmic reticulum, ER) are displayed in green color. In the middle, the red images (EX: 543 nm; EM: LP560 nm) of cells were stained with certain organelle dyes, e.g. propidium iodine (Vector Lab.) for nucleus; MitoTracker Orange CMTMRos (Invitrogen) for mitochondria; ER-Tracker Red (Invitrogen) for ER. The merged images are show in the bottom part. The white arrows indicate that certain targeted Met-cads co-localize with specific organelles within the same compartments of the cells (yellow color). Scale bar = 20 µm.
(TIF)

Figure S5 **The comparisons between Met-lead 1.59 and Met-cad 1.57.** Sensing key of Met-lead 1.59 was from part of PbrR (starting from the residue D between α3 and α4 helix in bold) [1]. And sensing key of Met-cad 1.57 was from part of CadR, in this study. The most different parts in sequences are after the 2^nd^ cysteine (denoted as star, after the end of the α5 helix) through the whole α6 helix (in red color).
(TIF)

Table S1 **The effects of reagents used (high K, TG, Nif, and 2-APB) on ratio value in** **Figure 5** **.** There’s no significant changes between the two experimental sets, i.e. before and 30 min. after reagent treatments).
(TIFF)

References S1 Supplemental Bibliography
(pdf)
