# Supplementary figures and images for: Live-Cell Dynamic Sensing of Cd2+ with a FRET-Based Indicator
Source: PLoS One. 2013 Jun 11;8(6):e65853. doi: 10.1371/journal.pone.0065853 (PMC3679114; doi:10.1371/journal.pone.0065853)

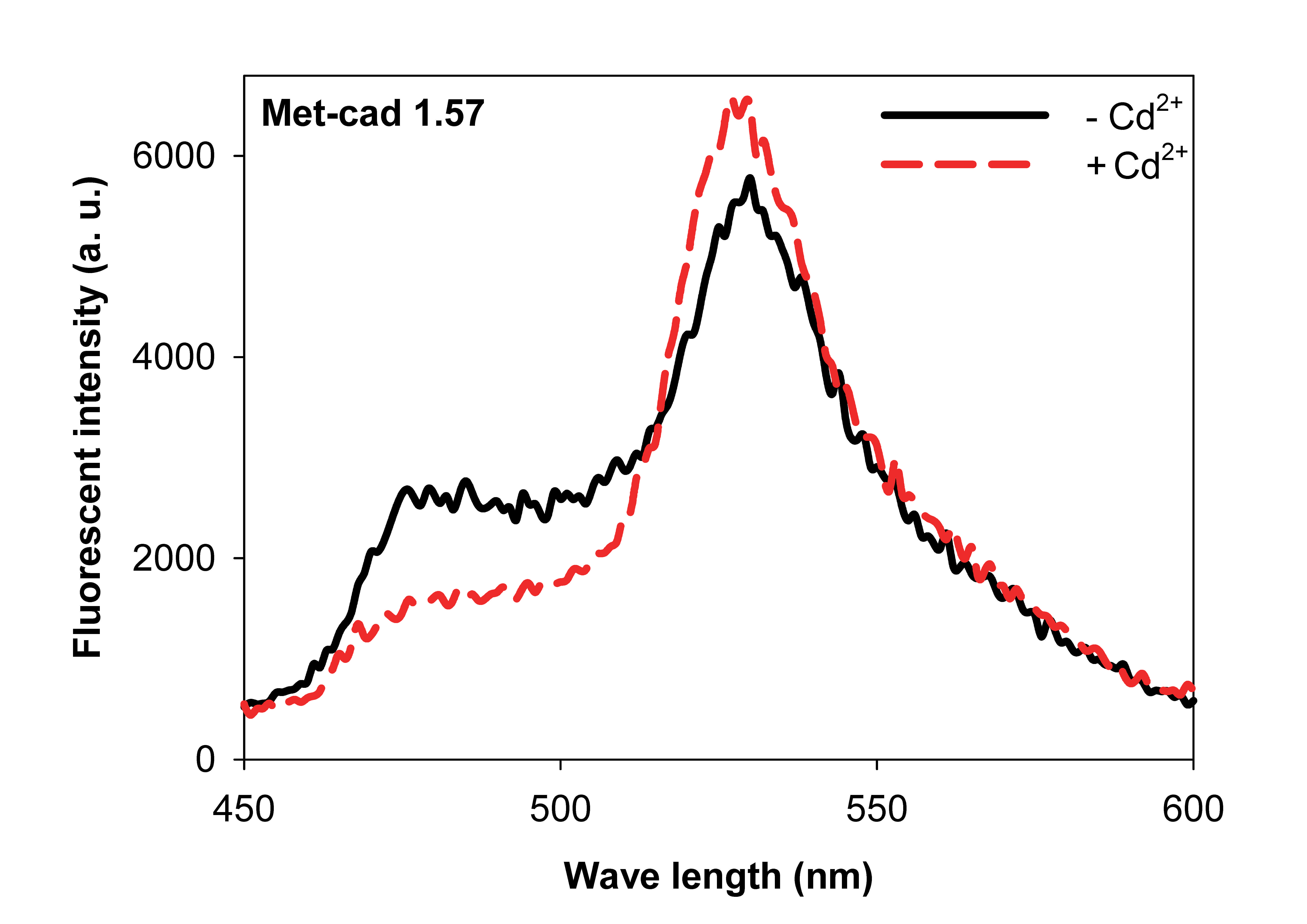

Supplement: Figure S1 — Spectral Pattern changes of mammalian Met-cad 1.57 between the control group [(–)Cd, solid line] and Cd [(+)Cd, 100 µM of Cd2+; red dashed line]. (TIFF) [file pone.0065853.s001.tiff]

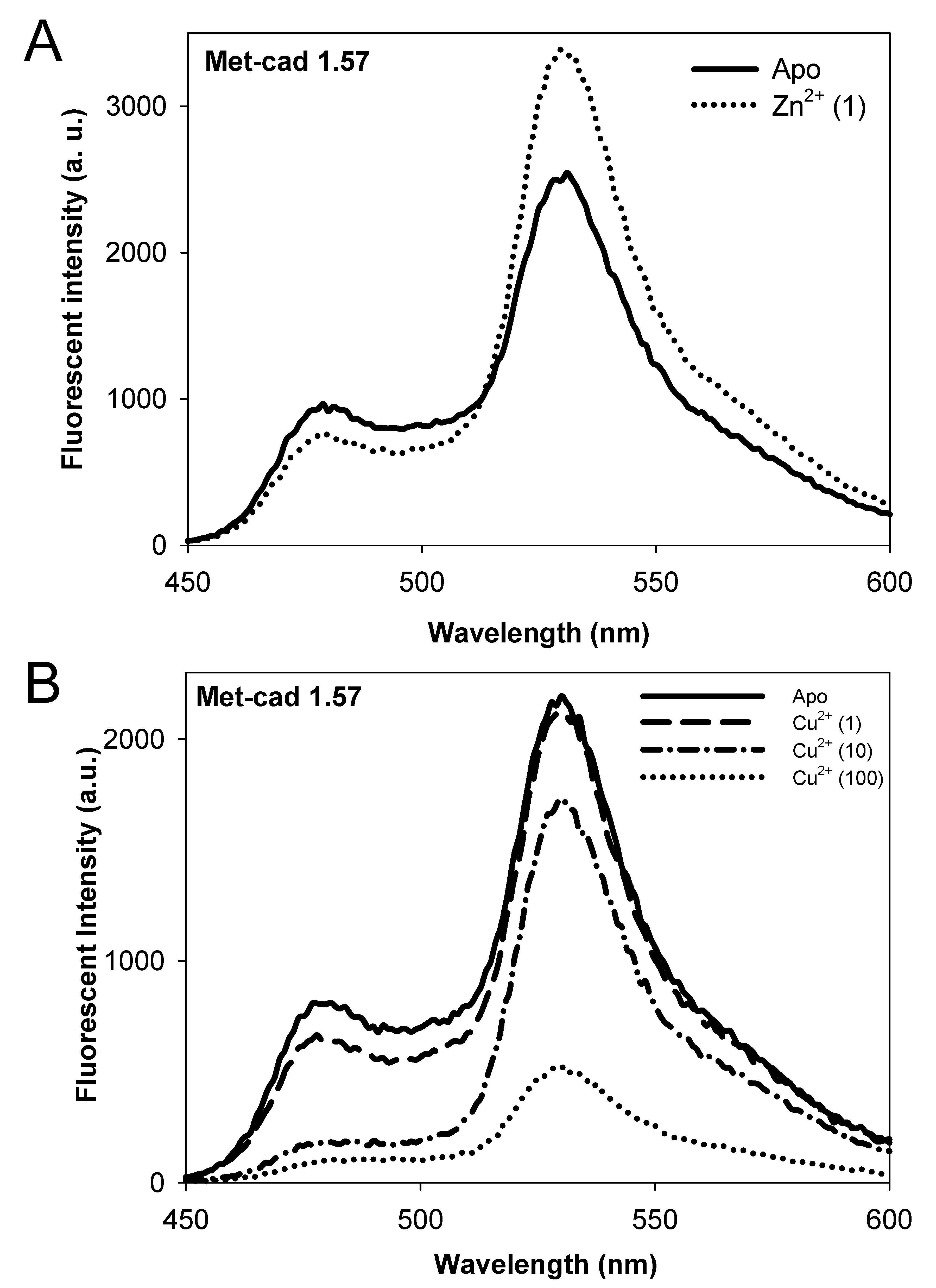

Supplement: Figure S2 — Effects of Zn2+ and Cu2+ on the spectral pattern of Met-cad 1.57. The spectral patterns of Met- cad 1.57 to Zn 2+ (A, 1 µM in dotted line) and to Cu2+ (B, 1 µM in dashed line; 10 µM in dashed-dotted line; 100 µM in dotted line) compared with the control without specific ions (Apo in solid line). (TIF) [file pone.0065853.s002.tif]

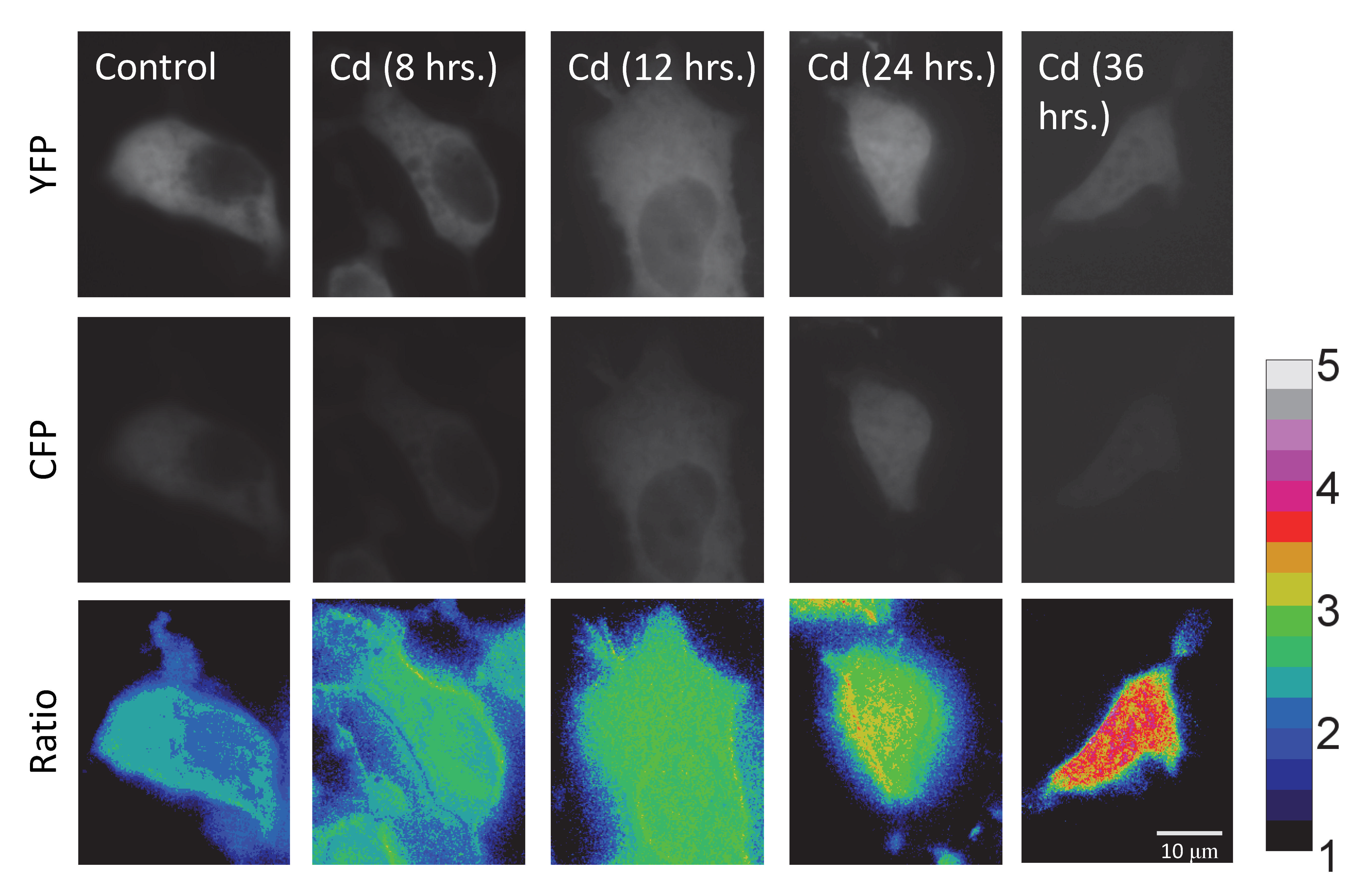

Supplement: Figure S3 — Long-term monitoring of intracellular Cd2+ content. The representative images of cells expressing Met-cad for monitoring intracellular content of Cd2+ are shown in YFP (top), CFP (middle), and ratio (bottom) with 8, 12, 24, and 36 hours of incubations (1 µM). The color bars show the ratio that ranges from 1 to 5. (TIF) [file pone.0065853.s003.tif]

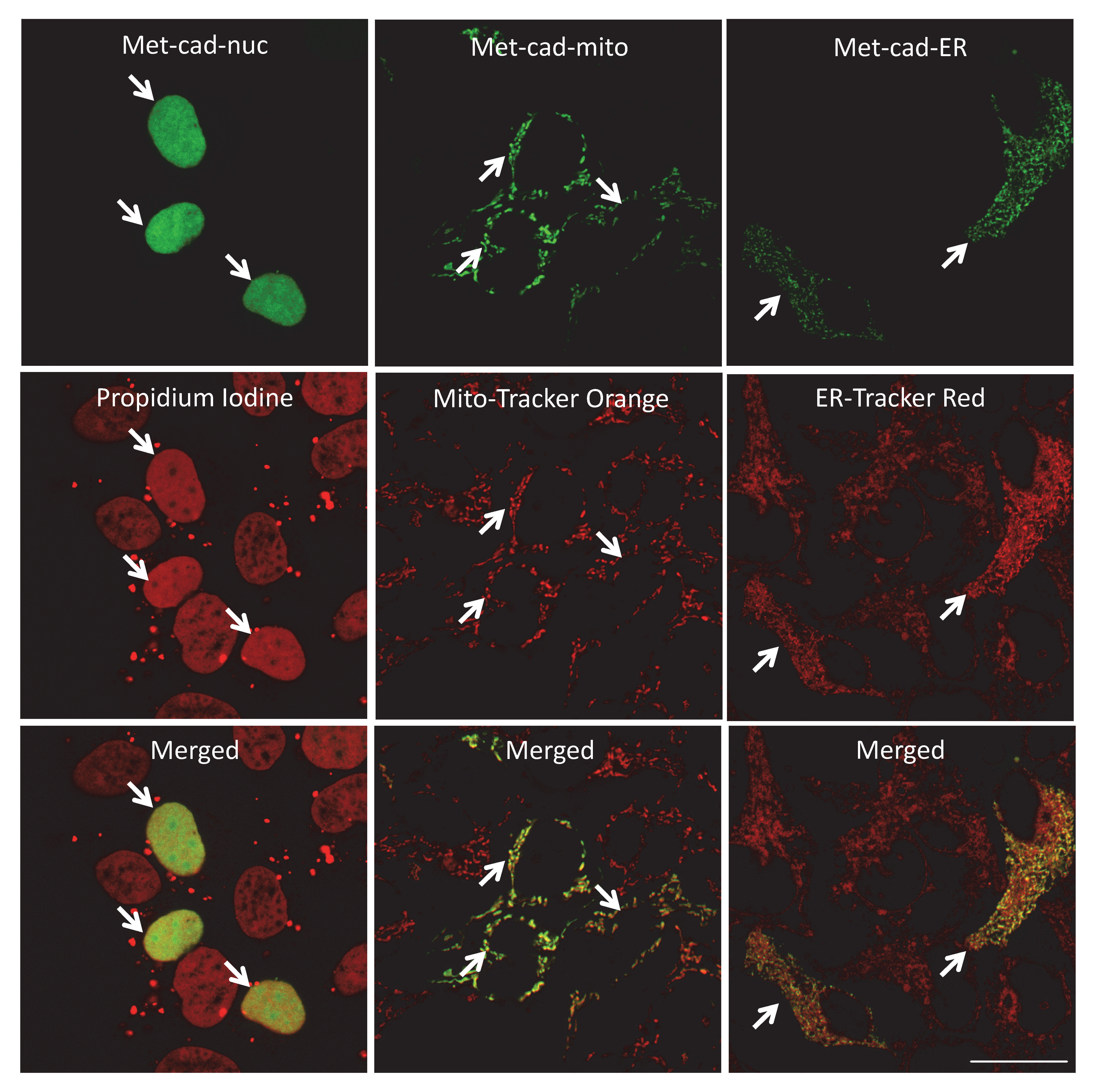

Supplement: Figure S4 — Sub-cellular targeting of Met-cads within cells. On top, the representative confocal images (LSM 5 Pascal, Zeiss, Germany, with a 63× oil objective, NA = 1.4, on an inverted microscope, Axiovert 200M) [1], [2] of Met-cads (EX: 458 nm; EM: LP475 nm) of cells transfected with various organelle-targeted versions (Met-cad-nuc for nucleus; Met-cad-mito for mitochondria; Met-cad-ER for endoplasmic reticulum, ER) are displayed in green color. In the middle, the red images (EX: 543 nm; EM: LP560 nm) of cells were stained with certain organelle dyes, e.g. propidium iodine (Vector Lab.) for nucleus; MitoTracker Orange CMTMRos (Invitrogen) for mitochondria; ER-Tracker Red (Invitrogen) for ER. The merged images are show in the bottom part. The white arrows indicate that certain targeted Met-cads co-localize with specific organelles within the same compartments of the cells (yellow color). Scale bar = 20 µm. (TIF) [file pone.0065853.s004.tif]

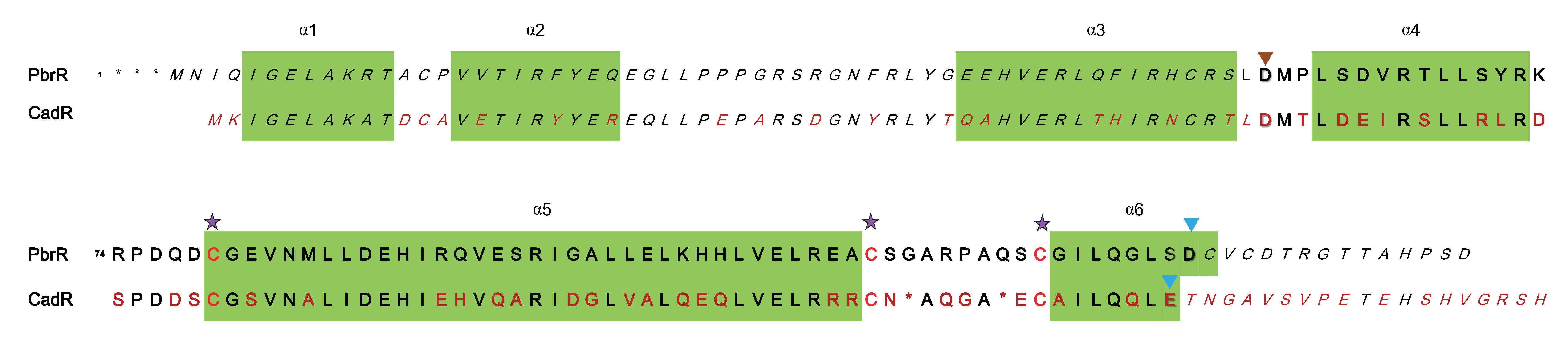

Supplement: Figure S5 — The comparisons between Met-lead 1.59 and Met-cad 1.57. Sensing key of Met-lead 1.59 was from part of PbrR (starting from the residue D between α3 and α4 helix in bold) [1]. And sensing key of Met-cad 1.57 was from part of CadR, in this study. The most different parts in sequences are after the 2nd cysteine (denoted as star, after the end of the α5 helix) through the whole α6 helix (in red color). (TIF) [file pone.0065853.s005.tif]

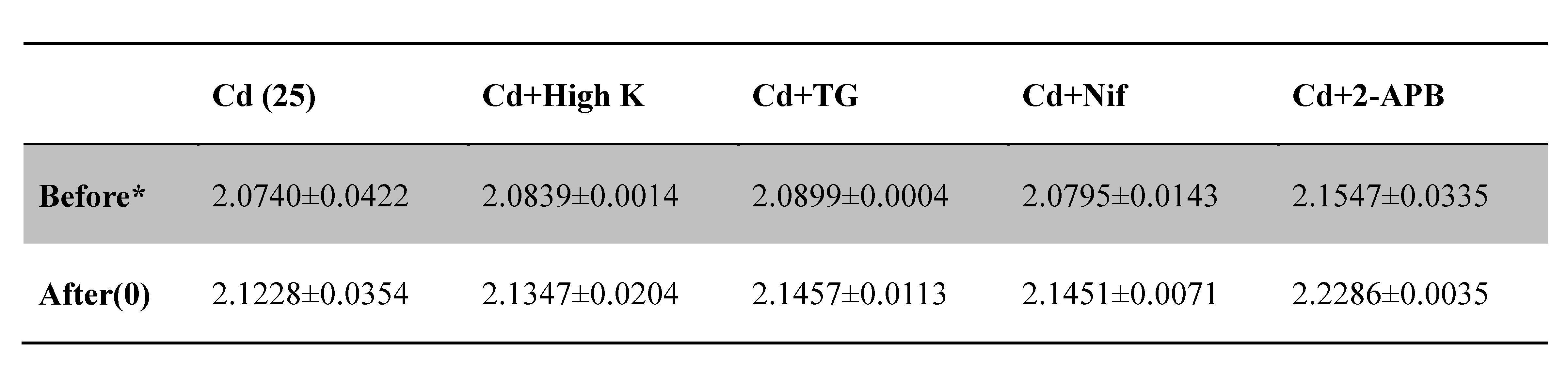

Supplement: Table S1 — The effects of reagents used (high K, TG, Nif, and 2-APB) on ratio value in Figure 5 . There’s no significant changes between the two experimental sets, i.e. before and 30 min. after reagent treatments). (TIFF) [file pone.0065853.s006.tiff]
