# Supplementary material for: Live-Cell Dynamic Sensing of Cd2+ with a FRET-Based Indicator
Source: PLoS One. 2013 Jun 11;8(6):e65853. doi: 10.1371/journal.pone.0065853 (PMC3679114; doi:10.1371/journal.pone.0065853)
Supplement: References S1 — Supplemental Bibliography (pdf) [file pone.0065853.s007.pdf]

References for supplementary information

1. Chuang TY, Au LC, Wang LC, Ho LT, Yang DM, Juan CC (2012) Potential effect of resistin on the ET-1-increased reactions of blood pressure in rats and Ca^{2+} signaling in vascular smooth muscle cells. J Cell Physiol 227: 1610-1618.
2. Huang PC, Chiu TY, Wang LC, Teng HC, Kao FJ, Yang DM (2010) Visualization of the Orai1 homodimer and the functional coupling of Orai1-STIM1 by live-cell fluorescence lifetime imaging. Microsco Microanal 16: 313-326.
